# Structural characterization of E22G Aβ_1–42_ fibrils *via*^1^H detected MAS NMR†

**DOI:** 10.1039/d4cp00553h

**Published:** 2024-04-26

**Authors:** Natalie C. Golota, Brian Michael, Edward P. Saliba, Sara Linse, Robert G. Griffin

**Affiliations:** a Department of Chemistry, Massachusetts Institute of Technology Cambridge MA 02139 USA rgg@mit.edu; b Francis Bitter Magnet Laboratory, Massachusetts Institute of Technology Cambridge MA 02139 USA; c Biochemistry and Structural Biology, Department of Chemistry, Lund University Lund SE 22100 Sweden

## Abstract

Amyloid fibrils have been implicated in the pathogenesis of several neurodegenerative diseases, the most prevalent example being Alzheimer's disease (AD). Despite the prevalence of AD, relatively little is known about the structure of the associated amyloid fibrils. This has motivated our studies of fibril structures, extended here to the familial Arctic mutant of Aβ_1–42_, E22G-Aβ_1–42_. We found E22G-Aβ_M0,1–42_ is toxic to *Escherichia coli*, thus we expressed E22G-Aβ_1–42_ fused to the self-cleavable tag N^Pro^ in the form of its EDDIE mutant. Since the high surface activity of E22G-Aβ_1–42_ makes it difficult to obtain more than sparse quantities of fibrils, we employed ^1^H detected magic angle spinning (MAS) nuclear magnetic resonance (NMR) experiments to characterize the protein. The ^1^H detected ^13^C–^13^C methods were first validated by application to fully protonated amyloidogenic nanocrystals of GNNQQNY, and then applied to fibrils of the Arctic mutant of Aβ, E22G-Aβ_1–42_. The MAS NMR spectra indicate that the biosynthetic samples of E22G-Aβ_1–42_ fibrils comprise a single conformation with ^13^C chemical shifts extracted from hCH, hNH, and hCCH spectra that are very similar to those of wild type Aβ_1–42_ fibrils. These results suggest that E22G-Aβ_1–42_ fibrils have a structure similar to that of wild type Aβ_1–42_.

## Abstract 2

Amyloid fibrils have been implicated in the pathogenesis of several neurodegenerative diseases, the most prevalent example being Alzheimer's disease (AD).

## Graphical abstract

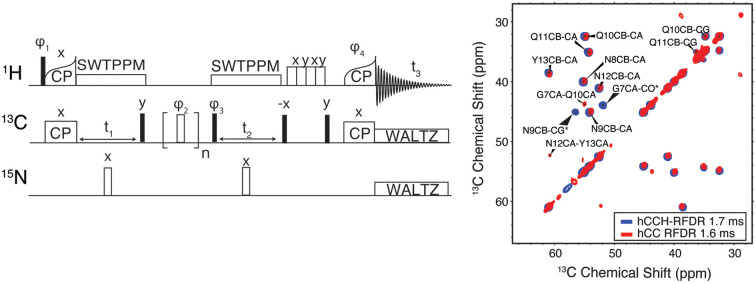

## 1. Introduction

The formation of amyloid fibrils by peptide misfolding and aggregation is implicated in the development of over 40 diseases, including Alzheimer's disease (AD), Parkinson's disease, and dialysis related amyloidosis (DRA). As of 2022,^1^ it is estimated that 1 in 9 adults over 65 have AD, which rises to 1 in 3 adults over 85. This figure is expected to increase as the US population continues to age. The aggregation of amyloid-β (Aβ) in the brain has been correlated with pathogenesis of AD, and, although not proven as the cause of AD, it remains a critical biomarker of the disease.^2–4^ Aβ occurs most frequently as a small protein of 40 or 42 residues and is derived by proteolytic cleavage of amyloid precursor protein (APP).^5–7^ While Aβ_1–42_ is expressed at lower levels than Aβ_1–40_, it is associated with greater cellular toxicity and is a major component of plaque found in AD patients.^8,9^ The development of aggressive, early onset forms of AD is associated with alterations to the APP. *In vitro*, enhanced aggregation dynamics are observed for mutations A21G, E22Δ, E22K, E22G, or D23N.^7,10–15^ The Arctic mutant, E22G, is characterized by the most rapid aggregation and fibrillization, thought to be driven by reduced electrostatic repulsive forces and decreased sidechain size.^16^ Structural studies of E22G-Aβ_1–40_ have revealed extensive polymorphism,^17,18^ while the characterization of E22G-Aβ_1–42_ has been limited to cryogenic electron microscopy.^19,20^ The first study examined two human brain protofilament extracts consisting of residues V12-V40 and E11-G37 of E22G, in addition to filaments from brains of mice with the Arctic mutation E22G.^19^ More recently, cryo-electron microscopy and cryo-electron tomography were used to determine the structure of Aβ from APP^NL-G-F^ mouse brains.^20^ In both cases, structural modifications were observed particularly in the case of the APP^NL-G-F^ mouse brain samples. However, to date, there has not been a MAS NMR study of E22G-Aβ_1–42_.

Since amyloid fibrils form a discontinuous solid phase of objects with high aspect ratio and are difficult to crystallize, they are not amenable to studies with either solution-state NMR or X-ray crystallography.^21^ However, because MAS NMR can address structural questions in such systems, it has become a valuable tool for probing dynamics, identifying polymorphism, and determining atomic resolution structures.^15,22,23^ In particular, development of dipole recoupling and fast magic angle spinning (MAS) methods^24^ have led to marked improvements in the resolution, sensitivity, and utility of biomolecular solid-state NMR.^25–28^ Applications to Aβ_1–42_ have revealed a monomorphic, dimeric structure of mirror image S-shaped monomers in which hydrophobic residues were isolated in the interior of the fibril core and ∼15 N-terminal residues form a dynamic tail.^22,23,29,30^ Only a few residues in the tail are observed in the MAS spectra, presumably because dynamics interferes with the ^1^H decoupling and broadens the signals.^31,32^ This structure represents one filament of two monomers per plane, while recent small angle scattering data reveal an elongated cross-section of four monomers per plane, representing two such filaments twisting together in a fibril.^33^ In addition, cryo-electron microscopy has been used to study amyloid fibrils and has proven to be particularly valuable in the study of brain derived samples.^19,23,34,35^

Only recently have applications of ^1^H-detected MAS methods to amyloid fibrils become more common. The advantages are an absolute sensitivity enhancement over conventional ^13^C detection^36^ and improved coherence lifetimes.^37,38^ The improved sensitivity has provided structural information about amyloid fibrils and membrane proteins beyond that available from directly detected ^13^C and ^15^N spectra.^39–45^ Conventional ^13^C detected methods have relied upon large sample volumes, moderate MAS frequencies (typically in the range *ω*_r_/2π ∼ 10–20 kHz), and radio frequency driven recoupling^46,47^ (RFDR) for homonuclear ^13^C–^13^C spin correlations and short-range resonance assignments. RFDR, a first order, zero quantum recoupling sequence that uses a single 180° pulse centered in the rotor period to recouple homonuclear dipolar couplings,^46–48^ has been frequently employed in probing amyloids, membrane proteins and viral particles.^49–53^ On the other hand, recent experiments combined homonuclear recoupling with MAS at *ω*_r_/2π ≥ 90 kHz to acquire ^1^H-detected multidimensional experiments on a few hundred micrograms of material. However, to date very few ^1^H detected studies were performed on fully protonated Aβ, demonstrating the need for continued development and application of dipolar recoupling at high spinning frequencies.^38,54^

In this study, we demonstrate the use of ^1^H detected NMR for high-resolution structural characterization of fully protonated GNNQQNY crystals and E22G-Aβ_1–42_ fibril samples. This work demonstrates the utility of the rigid crystal in optimizing the development of proton detected methods to the study of amyloidogenic proteins. A MAS frequency of *ω*_r_/2π = 90 kHz was sufficient to achieve ^1^H linewidths of ∼0.3 ppm in GNNQQNY using cross polarization single quantum correlation (CP-HSQC) ^1^H–^13^C and ^1^H–^15^N experiments. Furthermore, the hCCH-RFDR experiment was optimized on GNNQQNY, and we determined that the optimal mixing times for RFDR at *ω*_r_/2π = 90 kHz are comparable to those at *ω*_r_/2π = 20 kHz.

Biosynthetic expression of E22G-Aβ_1–42_ was difficult since the protein is toxic to *Escherichia coli* and leads to relatively poor yield of tag-free peptide.^16^ We therefore expressed E22G-Aβ_1–42_ as a fusion protein with the self-cleavable tag N^Pro^ in the form of its EDDIE mutant; that is, as EDDIE-E22G-Aβ_1–42_ (see the sequence provided below),^55^ which protects the cells by driving the expressed product to inclusion bodies. Nevertheless, the high surface activity of E22G-Aβ_1–42_ leads to high losses during purification, and the final yield of isotopically enriched E22G-Aβ_1–42_ were limited; thus, the improved sensitivity available from ^1^H-detected MAS NMR was essential for study of the E22G mutant.

The ^1^H–^13^C CP-HSQC spectra reveal a conformationally disordered A42, as previously observed in wild type Aβ_1–42_, and slight (≤1 ppm) chemical shift differences from wild type Aβ_1–42_. The ^1^H–^15^N CP-HSQC spectra have considerably broadened line shapes and reduced resolution, likely due to slight structural differences among fibrils due to the characteristic rapid fibril formation of Arctic mutant samples. ^15^N spectra are known to be sensitive to these effects since the ^15^N is directly involved in hydrogen bonding. Finally, we demonstrate the utility of homonuclear recoupling at *ω*_r_/2π = 90 kHz to determine that E22G-Aβ_1–42_ fibrils are largely monomorphic. The hCCH spectra, recorded using either RFDR or TOCSY mixing, confirm the small chemical shift perturbations observed in hCH spectra. Collectively, we use proton detected MAS NMR to conclude that E22G Aβ_1–42_ fibrils are monomorphic, with a fibril core structure similar to that of wild type Aβ_1–42_, in accordance with a recently published cryo-EM study.^19^

## 2. Materials and methods

### 2.1 GNNQQNY synthesis

Uniformly labeled ^13^C,^15^N-GNNQQNY was synthesized using solid phase peptide synthesis and purified *via* HPLC courtesy of the Swanson Biotechnology Center at the MIT Koch Institute. The sample purity was >98% as ascertained by mass spec analysis. Samples were crystallized at peptide concentrations < 10 mg mL^−1^ as previously described.^56,57^ The crystals were then packed into a 3.2 mm or 0.7 mm rotors using ultracentrifugation. The 3.2 mm rotors contained ∼20 mg of sample whereas the 0.7 mm rotor uses 0.5 mg.

### 2.2 Expression and purification of EDDIE-Aβ_1–42_ E22G

As mentioned above E22G-Aβ_M0,1–42_ is toxic to *Escherichia coli* and leads to poor yield of tag-free peptide.^16^ Therefore to express E22G-Aβ_1–42_ we employed a self-cleavable tag N^Pro^ in the form of its EDDIE mutant.^55^ This approach, which protects the cells by driving the expressed product to inclusion bodies, enables the isolation of Aβ peptides starting with Asp1 at the N-terminus,^58–60^ as well as the expression of other toxic peptides such as IAPP.^61^ The amino acid sequence of the expressed construct, EDDIE-E22G-Aβ_1–42_, is as follows:

MELNHFELLY KTSKQKPVGV EEPVYDTAGR PLFGNPSEVH PQSTLKLPHD RG**E̲D̲**DI**E̲**TTL RDLPRKGDCR SGNHLGPVSG IYIKPGPVYY QDYTGPVYHR APLEFFDE**T̲**Q F**E̲**E**T̲**TKRIGR VTGSDGKLYH IYV**E̲**VDG**E̲**IL LK**Q̲**AKRGTPR TLKW**T̲**RN**T̲**TN CPLWVTSC **D̲A̲E̲F̲R̲H̲D̲S̲G̲Y̲ E̲V̲H̲H̲Q̲K̲L̲V̲F̲F̲ A̲G̲D̲V̲G̲S̲N̲K̲G̲A̲ I̲I̲G̲L̲M̲V̲G̲G̲V̲V̲ I̲A̲**.

Here the EDDIE mutant is the first 158 residues^62^ with those that are bold and underlined being the mutated sites. The E22G-Aβ_1–42_ is entirely bold faced and underlined.

The gene construct was designed with *E. coli*-preferred codons in a pET3a plasmid (purchased from Genscript, Piscataway, New Jersey) and the fusion protein was expressed in *E. coli* BL21 DE3 PlysS star in M9 minimal medium with ^13^C_6_-glucose and ^15^NH_4_Cl as the sole carbon and nitrogen sources.

A cell pellet from 4 L of solution was sonicated 5 times in 80 mL 10 mM tris/HCl, 1 mM EDTA, pH 8.5 (buffer A) containing a trace of DNase, with centrifugation at 18 000*g* for 7 min between sonication steps. The inclusion body pellet after the 5th sonication was dissolved in 150 mL buffer A with 10 M urea and 1 mM DTT, by sonication and stirring. The resulting solution (*ca.* 9.4–9.7 M urea) was diluted with 200 mL buffer A with 1 mM DTT (thus yielding *ca.* 4 M urea) and loaded onto 2 × 20 mL DEAE-sepharose FF columns (GE Healthcare (now Cytiva)) in tandem, equilibrated in buffer A with 4 M urea and 1 mM DTT. The columns were washed with 100 mL buffer A with 4 M urea and 1 mM DTT, and eluted with a 0–0.4 M NaCl gradient in the same buffer. Fractions containing EDDIE-E22G-Aβ_1–42_ were diluted 15 times with 1 M tris, 1 mM EDTA, 5 mM DTT, pH 7.9 in glass bottles and left at 4 °C for 48 h, total volume 1.0 L. During this time EDDIE slowly folded leading to auto-cleavage and release of E22G-Aβ_1–42_. The solution was then dialyzed in 3.5 kDa MW cutoff dialysis bags (boiled 4 times in Millipore water before use) against a total of 30 L of 5 mM tris/HCl, 0.5 mM EDTA, pH 8.5, in three shifts (10 L per shift). The dialyzed solution was supplemented with 50 g Q-sepharose big beads (GE Healthcare, equilibrated in buffer A) and incubated for 0.5 h in the cold room with occasional stirring using a glass rod. The beads were collected on a Büchner funnel and washed with 200 mL buffer A. E22G-Aβ_1–42_ was eluted in buffer A with 50 mM NaCl, 8 fractions of 50 mL each. The fractions were lyophilized, dissolved 2-by-2 in 10 mL 6 M GuHCl and isolated from any residual *E. coli* proteins, aggregates, and small molecule contaminants by size exclusion chromatography (SEC) in 20 mM sodium phosphate, 0.2 mM EDTA, 0.02% (w/v) NaN_3_, pH 8.5 using a Superdex 75 26/600 column. The eluted fractions were monitored by UV absorbance and SDS PAGE with Coomassie staining. Fractions corresponding to the center of the E22G-Aβ_1–42_ monomer peak were pooled in a glass bottle, pH adjusted to 8.0 by adding NaH_2_PO_4_ and the pool was incubated quiescently at 37 °C. Monomer from each of the four rounds of SEC were added to the same bottle to propagate the morph formed in the first of the four aliquots.

The formation of fibrils was validated for a small sample using thioflavin T fluorescence in a PerkinElmer LS50B fluorescence spectrometer, as compared with the thioflavin T fluorescence in buffer. The morphology of the fibrils, as revealed by cryo-TEM, is indistinguishable from that of the wild type peptide, displaying a double-filament structure with a short twist distance between the apparent cross-over points.^16^

We also purified one aliquot of E22G-Aβ_1–42_ after expression of NT*-E22G-Aβ_1–42_ (synthetic gene with *E. coli*-preferred codons in pET3a plasmid purchased from Genscript) in *E. coli* BL21 DE3 PlysS star in 1 L M9 minimal medium with ^13^C-glucose and ^15^NH_4_Cl as the sole carbon and nitrogen sources using the published protocol.^63^ After the final SEC, we mixed this aliquot into the same solution as described above.

The samples were finally packed into 0.7 mm diameter rotors using ultracentrifugation. Tryptic digest and intact mass spectrometry, as described in the ESI† (Fig. S1, S2 and Table S1), were used to confirm the desired sequence with 97.3% isotopic enrichment and absence of Aβ_1–42_ wild type fibrils. The 0.7 mm rotors used to record the E22G spectra contain 0.5 mg peptide.

### 2.3 Magic angle spinning nuclear magnetic resonance

^1^H detected spectra were acquired at a static field of 18.8 T (800 MHz for ^1^H) with a three channel (HCN) 0.7 mm Bruker MAS probe. The spinning frequency of *ω*_r_/2π = 90 kHz was regulated using a MAS 3 controller and temperature was maintained at 273 K using a Bruker cooling unit. The Rabi fields (and 90° pulse durations) for ^1^H, ^13^C, and ^15^N were 133 kHz (1.875 μs), 83 kHz (3 μs), and 83 kHz (3 μs), respectively.

Dipolar based CP-HSQC spectra were recorded using the pulse sequence previously described using the zero-quantum cross polarization condition at nutation frequencies near (5*ω*_r_/4) and (*ω*_r_/4), for ^1^H and ^15^N or ^13^C, respectively.^44^

**Fig. 1:**
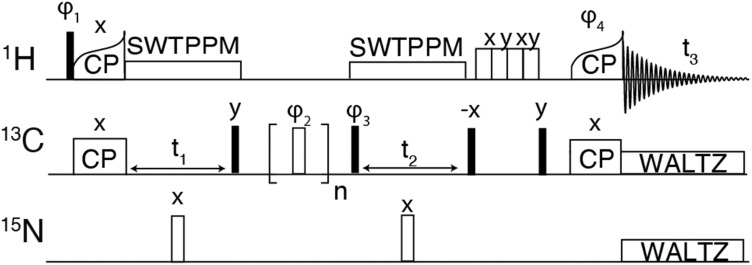
Pulse sequence for 3-dimensional hCCH experiment using RFDR for carbon–carbon mixing. The phase cycle *φ*_1_ was [*y*, −*y*], *φ*_2_ was XY-32[*x*, *y*, *x*, *y*, *y*, *x*, *y*, *x*, *y*, −*x*, *y*,−*x*, −*x*, *y*, −*x*, *y*, −*x*, −*y*, −*x*, −*y*, −*y*, −*x*, −*y*, −*x*, −*y*, *x*, −*y*, *x*, *x*, −*y*, *x*, −*y*], *φ*_3_ was [*x*, *x*, −*x*, −*x*], *φ*_4_ was [4*x, 4*−*x*]. The receiver phase cycle was [*x*, −*x*, −*x*, *x*, −*x*, *x*, *x*, −*x*].

For the hCCH experiments, the ^1^H–^13^C CP was performed using a tangential ramp on the proton RF power and a linear 10% ramp was used for the ^13^C–^1^H CP. For hCCH-TOCSY, WALTZ-16 was used with an RF field of ∼22.5 kHz corresponding to (*ω*_r_/4). For hCCH-RFDR experiments, illustrated in Fig. 1, the π-pulse width was 2.5 μs corresponding to an RF field of 200 kHz. At *ω*_r_/2π > 30 kHz, ^1^H decoupling is not required during the RFDR mixing period as the mixing pulses recouple and decouple simultaneously.^53^ Spectra were apodised using 60° shifted squared sine bells or Gaussian apodization and zero filled to at least twice the number of points in the indirect dimension. Details of the acquisition parameters are provided in Table S2 (ESI†).

In all ^1^H detected experiments, swept-low power TPPM (SW-TPPM) at an RF field amplitude of *ω*_^1^H_/2π ∼ 24 kHz was used for proton decoupling during *t*_1_ and *t*_2_, and WALTZ-16 was applied to ^13^C and ^15^N at *ω*_^1^H/^1^C_/2π = 10 kHz, respectively.^64,65^ Water suppression was achieved through the MISSISSIPPI pulse sequence without homospoil gradients,^66^ using a 24 kHz RF field and 200 ms pulse duration. Conventional hCC-RFDR spectra were acquired at a field of 18.8 T (800 MHz for ^1^H) with a three channel (HCN) 3.2 mm Bruker MAS probe at a MAS rate of 20 kHz and a temperature of 273 K. For the 3.2 mm probe, Rabi fields were 83 kHz for ^1^H and ^13^C. RFDR mixing was performed using a 180° pulse width of ∼6 μs and a CW proton decoupling field of 83 kHz.

## 3. Results and discussion

In order to validate the pulse experiment shown in Fig. 1, we use the amyloidogenic peptide GNNQQNY which is part of the terminal domain of Sup35p, an amyloid forming yeast prion protein.^56^ Early studies found the monoclinic microcrystals of GNNQQNY, as used herein, have amyloidogenic properties including binding of Congo red and formation of β-sheet and steric zipper structures. High resolution X-ray crystal structures have been obtained, in addition to ^13^C and ^15^N assignments *via* MAS NMR.^57,67–70^ The monoclinic crystals are rigid and offer an amyloidogenic system with which to optimize high resolution protein spectroscopic methods.

**Fig. 2:**
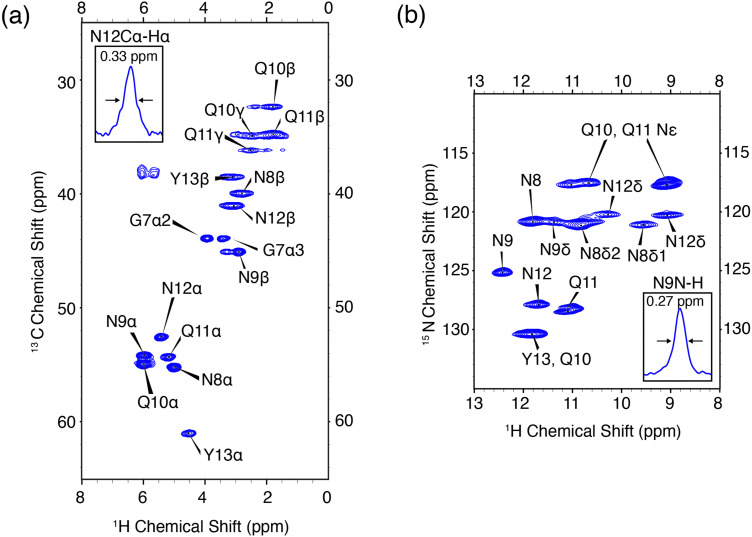
High resolution ^1^H detected CP-HSQC spectra of nanocrystalline U-^13^C,^15^N-GNNQQNY at *ω*_r_/2π = 90 kHz, *ω*_^0^H_/2π = 800 MHz. (a) ^1^H–^13^C correlations. (b) ^1^H–^15^N correlations. The mixing times and other spectral acquisition parameters are provided in Table S2 (ESI†).

The ^1^H–^13^C and ^1^H–^15^N heteronuclear correlation spectra of monoclinic crystals of GNNQQNY and the associated proton assignments are shown in Fig. 2. These are the first ^1^H detected experiments of GNNQQNY and linewidths of 0.33 ppm and 0.27 ppm were obtained for the Cα proton of N12 and the NH proton of N9, respectively. The degeneracy of amino acids in this sample results in spectral overlap, reducing the effective resolution of many resonances. Typically, proton linewidths were less than 0.3 ppm for individual resonances, where all linewidths are reported without apodization. The narrow line shapes in spectra of the fully protonated sample are indicative of the highly ordered crystal structure of GNNQQNY, while further reduction in linewidth is expected with application of higher MAS rates.

The hCH experiment was then used as a building block for the hCCH-RFDR experiment initially implemented on GNNQQNY, using the pulse sequence shown in Fig. 1. A MAS frequency of *ω*_r_/2π = 90 kHz and *ω*_^1^C_/2π = 200 kHz RFDR pulses were sufficient to recouple ^13^C–^13^C and decouple ^1^H–^13^C. Thus, conventional ^1^H decoupling during the RFDR mixing period was not required. At a MAS frequency of *ω*_r_/2π = 90 kHz, the theoretical description describing RFDR requires accounting for the finite pulse effects, given that the 2.5 μs 180° pulse used occupies a considerable fraction of the 11.11 μs rotor period. For a further description of finite pulse RFDR, we refer the reader to the original paper by Bennett, *et al.* where finite pulses were initially discussed.^47^

**Fig. 3:**
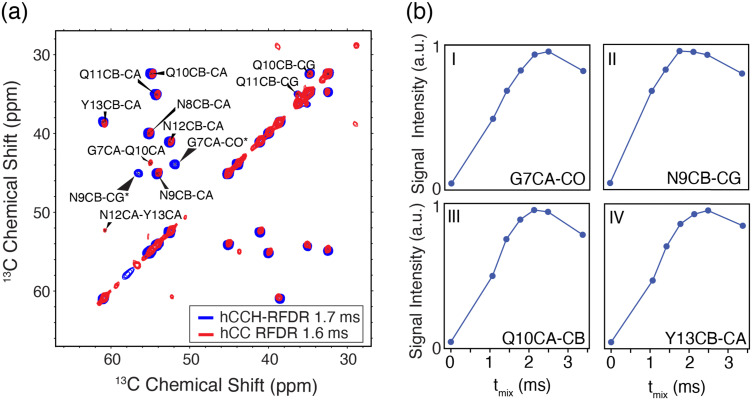
hCCH-RFDR spectra of nanocrystalline GNNQQNY. (a) The ^13^C–^13^C correlations of hCCH-RFDR (red) and hCC RFDR (blue) of U[^13^C,^15^N]-GNNQQNY crystals. The hCCH-RFDR spectrum was acquired at *ω*_r_/2π = 90 kHz using a mixing time of 1.7 ms. The conventional 2-dimensional hCC-RFDR spectrum was acquired at *ω*_r_/2π = 20 kHz MAS using a mixing time of 1.6 ms. The N9Cβ-Cγ* and G7Cα-C_0_* peaks are folded. (b) Signal intensity as a function of RFDR mixing time for hCCH-RFDR. For each contact in I–IV, the normalized signal intensity is plotted.

As shown in Fig. 3(A), the hCCH-RFDR at *ω*_r_/2π = 90 kHz reproduces all intra-residue correlations that are expected in a one-bond RFDR. While numerous other heteronuclear and homonuclear correlation methods have been developed for ^1^H detected MAS NMR, the direct comparison of hCCH to hCC spectra acquired at 20 kHz MAS is a valuable spectroscopic tool for spectral fingerprinting of limited quantity samples. However, we note that in the hCCH-RFDR spectrum, the very weakly detected inter-residue correlations of N12Cα-Y13Cα and G7Cα-Q10Cα were not visible. These longer-range correlations may be more effectively probed at high spinning frequencies by other homonuclear correlation schemes or with additional transients per point. In the present spectrum, 8 scans per point were acquired for the hCCH-RFDR leading to a total experiment time of 2 days and 15 hours.

As shown in Fig. 3(B), most residues have achieved maximum signal intensity between 1.7 and 2.5 ms, which is comparable to optimal mixing times at lower MAS frequencies, such as *ω*_r_/2π = 20 kHz. The longest optimal mixing times are observed for the G7 and Y13 residues, which are excluded from the steric zipper core interface between peptide dimers. Thus, the hCCH-RFDR detected spectra of GNNQQNY at high resolution permitted us to optimize spectral fingerprinting and determine optimal mixing times, which are comparable to those canonically used at lower MAS frequencies.

We then employed the ^1^H detected experiments to characterize E22G-Aβ_1–42_ by MAS NMR. As detailed above, the E22G-Aβ_1–42_ mutant was expressed using a fusion construct with the EDDIE mutant of N^pro^ expressed in *E. Coli* for uniform ^13^C and ^15^N isotopic enrichment. However, due to the very high surface activity of the free peptide, only limited sample quantities could be purified after cleveage, and as such, proton detected MAS NMR were essential for reasons of sensitivity. The expression protocol was previously validated with for example wild type Aβ_1–42_^60,71^ and cryo-TEM revealed the morphology of the E22G-Aβ_M1–42_ fibrils to be indistinguishable from the wild type Aβ_M1–42_ fibrils, with both fibrils displaying a double filament structure with short twist distance between apparent cross over points.^16^

**Fig. 4:**
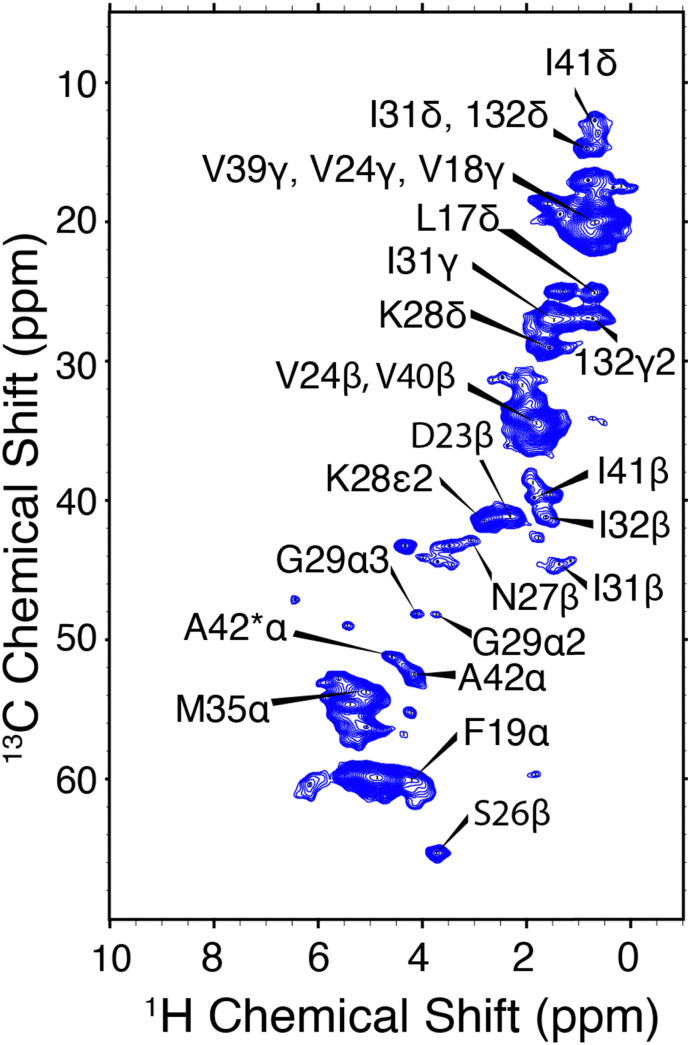
hCH correlations of E22G-Aβ_1–42_ amyloid fibrils. The assigned chemical shifts are consistent with a structure similar to Aβ_1–42_. *ω*_r_/2π = 90 kHz and *ω*_^0^H_/2π = 800 MHz. Mixing times and other spectral acquisition parameters are tabulated in Table S2 (ESI†).

CP-HSQC spectra were acquired for E22G-Aβ_1–42_ and illustrated in Fig. 4. The hCH spectra showed only small chemical shift perturbations from the previously published wild type Aβ_M1–42_ fibril spectra.^38^ We note an overall reduction in the spectral resolution of the E22G sample acquired at *ω*_r_/2π = 90 kHz and *ω*_^1^H_/2π = 800 MHz, relative to the wild type sample at *ω*_r_/2π = 111 kHz and *ω*_0_/2π = 1 GHz.^38^ Two resonances were assigned to the A42 ^13^Cα in both the E22G spectrum (Fig. 4) and the previously studied wild type sample.^23,72^ This peak doubling, which is attributed to two conformations of the A42 residue, was observed in wild type Aβ_M1–42_ only at *ω*_r_/2π = 111 kHz and was not observed at *ω*_r_/2π = 20 kHz. E22G-Aβ_1–42_ cross peak assignments (Fig. 4) were rapidly obtained due to small chemical shift perturbations (Fig. 6) in the hCH spectra of E22G-Aβ_1–42_ relative to previously obtained assignments of wild type samples. The shared presence of two conformations of the C-terminal segment in both wild type and E22G-Aβ_1–42_ initially suggested a conserved C-terminal structural fold. There are several unassigned, narrow resonances that could also be attributable to dynamic residues in the N-terminal segment. These observations underscore the importance of developing high spinning frequency MAS spectroscopy alone, or in combination with low temperature MAS, to study the N- and C-terminal segment structures and dynamics.

The hNH spectrum of E22G-Aβ_1–42_ (Fig. S3, ESI†) shows a reduction in resolution, the only cross peak that can presently be unambiguously assigned being due to A42. The lower resolution of the ^15^N spectral dimension has been observed previously in Aβ fibrils,^54^ and is likely due to structural heterogeneity, which results from the rapid fibril formation; thus, many studies of Aβ amyloid do not report ^15^N spectra. It is possible that polymorphism, sample impurities, and dynamics also contribute to the broadening.

To probe the role of potential polymorphism beyond the likely structural doubling of A42, we employed the hCCH-RFDR and hCCH-TOCSY experiments. hCCH-RFDR offers a direct spectral comparison to the wealth of previously acquired hCC spectra of WT Aβ_1–42_ in the literature.^22,23,72^ Despite the common occurrence of hCC-RFDR, the hCCH-RFDR has to date been sparsely applied in fully protonated samples^54,73^ or in selectively deuterated samples.^74^ Instead, RFDR is most often incorporated into hCHH or hNHH pulse sequences which offer increased sensitivity and the potential for longer range quantitative ^1^H–^1^H distance measurements.^38,39,45,75^ However, in fully protonated samples the continued development of high spinning frequency MAS rotors and pulse sequence methodology is required.

**Fig. 5:**
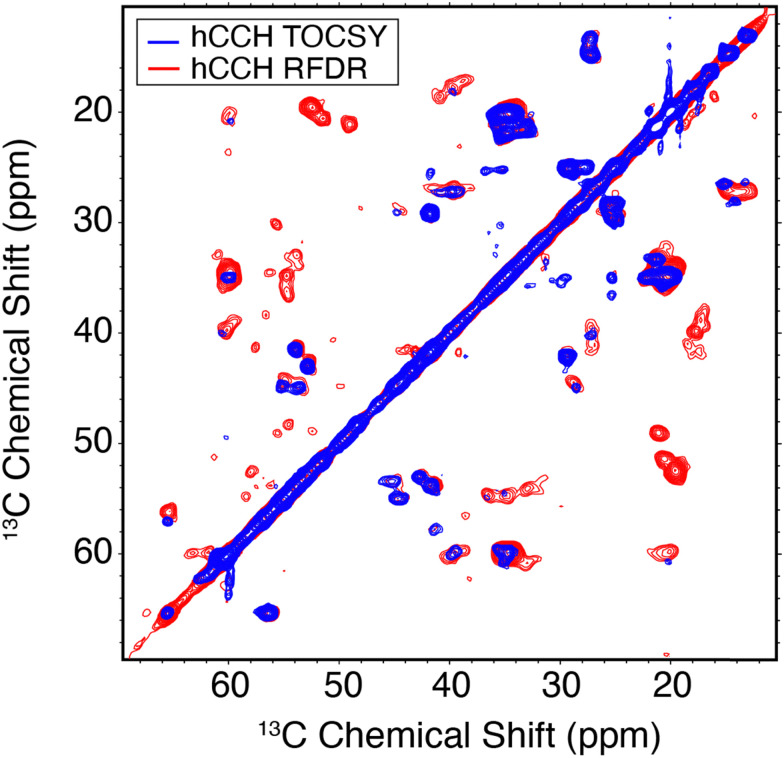
^13^C–^13^C hCCH spectra of E22G-Aβ_1–42_ amyloid fibrils using TOCSY (red) or RFDR (blue) for carbon homonuclear mixing. Mixing times and other spectral acquisition parameters are tabulated in Table S2 (ESI†).

A comparison between the hCCH-RFDR and hCCH-TOCSY spectra is illustrated in Fig. 5. The TOCSY based hCCH was described previously, with mixing largely mediated by ^13^C–^13^C scalar couplings^43^ and is based on total through bond correlation pulse sequences.^76^ As such, MAS rates *ω*_r_/2π > 90 kHz are expected to improve the performance of the pulse sequence on a dynamic sample such as the E22G-Aβ_1–42_ fibrils. Because it relies on dipolar rather than *J*-couplings the performance of RFDR is expected to be superior to that of TOCSY when a large chemical shift difference is present. Thus, in Fig. 5 we observe more cross peaks with the hCCH-RFDR experiment. For both pulse sequences, the C–C projection of the hCCH experiment displays improved resolution over the hCH detected experiments, although site specific resolution is still not achieved with 2D spectra in this sample. Thus, higher MAS frequencies, access to higher magnetic fields and higher dimensional spectroscopy remain critical steps for complete structural studies of pathologically relevant Aβ fibrils. Despite these limitations, several important preliminary biological conclusions can be drawn using the spectra presented here.

In Aβ_1–42_ fibrils serine residues, S8 and S26, were previously shown to be sensitive to polymorphism.^22,72^ Specifically, when polymorphs are present, then the Cα–Cβ cross region (∼55/57 ppm in Fig. 5) shows multiple peaks. In the present E22G-Aβ_1–42_ sample, the S26 Cα–Cβ correlation was observed as a single resonance, with ∼1 ppm in chemical shift difference relative to wild type Aβ_1–42_. Aside from the structural doubling of A42Cα, no further resonance doubling was observed. Compared to wild type Aβ_1–42_ RFDR fingerprint spectra, E22G-Aβ_1–42_ is similarly monomorphic. This observation is further supported by the single S26β hCH cross peak present in Fig. 4.

In a previous study^17^ of E22G-Aβ_1–40_, which employed a sample prepared synthetically, rather than biosynthetically, six specific residues (F19,A21, I32, L34, V36, and G38) in the core of the fibril structure were ^13^C/^15^N labeled. The spectra were well resolved and clearly showed four different cross peaks for the labeled residues. Although it is difficult to discern the reasons for the polymorphism in samples used in other studies, two factors, the purity of synthetic samples and the possibility of racemization of the three His residues in Aβ, are discussed in the literature.^77–79^ And in a separate investigation,^18^ it was shown that folding of E22G-Aβ_1-40_ fibrils could be enhanced with seeds of Aβ_1–42_, whereas WT Aβ_1–40_ was not affected. The spectra in these experiments obtained from synthetic samples (labeled as G22, V24, A30 and I31) displayed two-fold polymorphism in two of the four selectively labeled sites. Thus, polymorphism is observed in synthetic Aβ_1–40_ samples, *e.g.* E22G-Aβ_1–40_, whereas we did not detect it in E22G-Aβ_1–42_ prepared using biosynthetic methods. Nevertheless, these experiments suggested the dominant form of the Arctic mutant may adopt β-strand conformations similar to the wild type Aβ_1–42_, and this hypothesis is further supported by the MAS NMR data presented here.

**Fig. 6:**
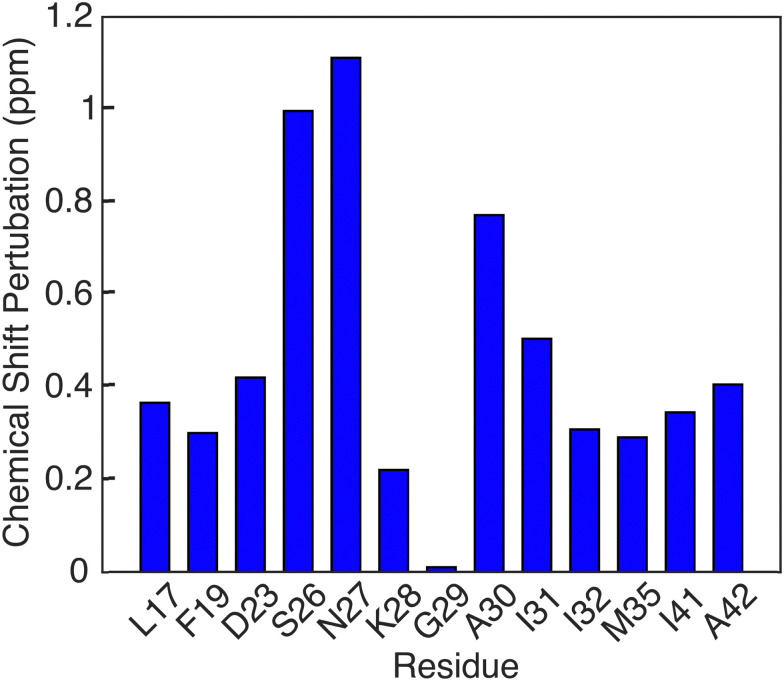
Chemical shift pertubations of E22G Aβ_1–42_ fibrils relative to wild type Aβ_1–42_ fibrils found in [ref. 23, 38, 50].

Another notable feature in the present E22G-Aβ_1–42_ is the minimal chemical shift perturbations associated with the K28 residue (∼0.2 ppm), suggesting that the K28-A42 salt bridge observed previously in wild type Aβ_1–42_ is likely conserved in E22G-Aβ_1–42_. A detailed compilation of the shifts in E22G-Aβ_1–42_ and Aβ_1–42_ is presented in Tables S3 and S4 (ESI†) In addition, the ^15^N–^1^H correlation spectra (Fig. S3, ESI†) also show a doublet at (135.5 ppm,9.5 ppm) in excellent agreement with the shifts observed in Aβ_1–42_.^23,72^ The figure allows a comparison of the NH spectra of E22G-Aβ_1–42_ with those of WT Aβ_1–42_. Similar salt bridges are present in the E22Δ-Aβ_1–39_ mutant structure linking E3 and K28.^15^ We do note that the largest observed chemical shift perturbations occur in adjacent residues S26 and N27, but they remained limited to ∼1 ppm. This supports the hypothesis that the mutation conserves fibril core features while inducing electrostatic perturbations that would make the wild-type fibril fold even more stable for the E22G mutant due to reduced electrostatic repulsion.^16^ As for the wild-type, the main driving force for fibril formation of E22G-Aβ_1–42_ are thus hydrophobic interactions due to burial of a large number of non-polar residues in the fibril core and the associated increase in entropy of the released water molecules.^16^ Overall, the core residues of the E22G-Aβ_1–42_ fibril display minimal chemical shift perturbations, on the order of ≤1 ppm, as illustrated in Fig. 6.

## 4. Conclusion

We present the initial ^1^H-detected MAS NMR results on two amyloidogenic systems, GNNQQNY monoclinic crystals and E22G-Aβ_1–42_ fibrils. Linewidths typically <0.3 ppm were observed in CP-HSQC spectra of the rigid peptide GNNQQNY. This high-resolution model system was employed to optimize hCCH-RFDR experiments, and an optimal mixing period in this system was observed to be ∼2 ms, on par with mixing periods used at lower MAS frequencies. These methods were then extended to the study of E22G-Aβ_1–42_ fibrils. The spectral resolution was reduced relative to wild type Aβ_1–42_, indicating an increase in sample heterogeneity perhaps due to the rate at which the fibrils are formed. The use of MAS at *ω*_r_/2π = 90 kHz confirmed the observation of dynamics in the A42 residue as observed also in wild type samples, indicating the C-terminus is not strongly modulated by the E22G mutation. hCCH-RFDR demonstrated the sample to be largely monomorphic, in contrast to studies of synthetic E22G-Aβ_1–40_ in which multiple polymorphs were observed. Overall, the chemical shift perturbations observed across numerous residues are <1 ppm between the previously reported wild type Aβ_1–42_ and the present E22G-Aβ_1–42_. This result suggests a conserved core fibril structure, in accordance with recent cryoEM results. Taken as whole, this study presents further evidence that the Arctic mutant has a propensity for adopting Aβ_1–42_ like fibril features as previously observed in Aβ_1–40_ fibrils of E22G mutant. In the future, the use of dynamic nuclear polarization and access to increased MAS rates and higher fields could aid in the determination of the complete three-dimensional structure of the sample quantity limited E22G mutant of Aβ_1–42_.

## Conflicts of interest

There are no conflicts to declare.

## Supplementary Material

CP-026-D4CP00553H-s001
