# Supplementary material for: Structural characterization of E22G Aβ1–42 fibrils via1H detected MAS NMR
Source: Phys Chem Chem Phys. 2024 Apr 26;26(20):14664–74. doi: 10.1039/d4cp00553h (PMC11110645; doi:10.1039/d4cp00553h)
Supplement: CP-026-D4CP00553H-s001 [file CP-026-D4CP00553H-s001.pdf]

Electronic Supplementary Material (ESI) for Physical Chemistry Chemical Physics.
This journal is © the Owner Societies 2024

Supplementary Information

Structural Characterization of E22G $A\beta_{1-42}$ Fibrils via ^{1}H detected MAS NMR

Natalie C. Golota[1,2], Brian Michael[1,2,5], Edward P. Saliba[1,2,6], Sara Linse[3], and Robert G. Griffin[1,2*]

[1] Department of Chemistry, Massachusetts Institute of Technology, Cambridge, MA, 02139, USA.

[2] Francis Bitter Magnet Laboratory, Massachusetts Institute of Technology, Cambridge, MA, 02139, USA

[3] Biochemistry and Structural Biology, Department of Chemistry, Lund University, Lund SE 22100, Sweden

* Corresponding author. Email: rgg@mit.edu

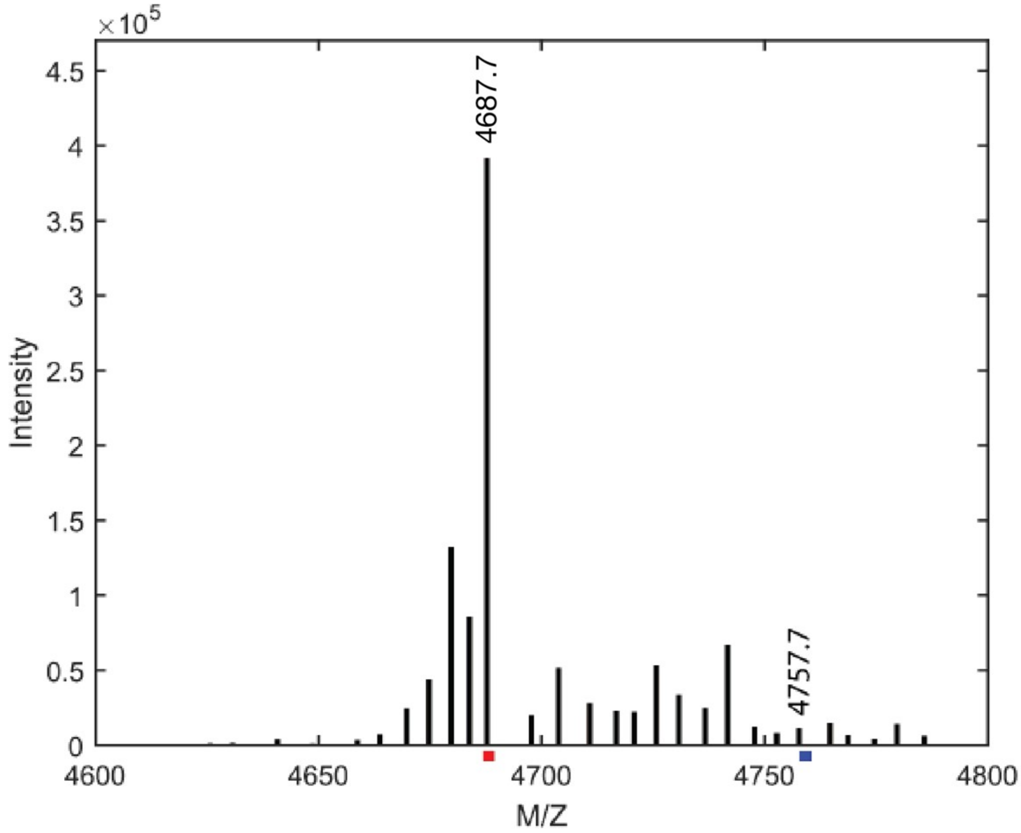

Figure S1: Intact MS of A$\beta_{1\text{-}42}$ E22G Sample. The predominant peak corresponding to 4687.7 m/z is the E22G-A$\beta_{1\text{-}42}$ corresponding to ~ 97.3% isotopic enrichment. The distribution of small peaks can correspond to sample impurities or between 95-100% isotopic enrichment of the sample. The range of 97%-98% isotopic enrichment is provided in the red bar for E22G-A$\beta_{1\text{-}42}$ or the blue bar for the wild type A$\beta_{1\text{-}42}$. Given the presence of a small peak minimally outside the range of 97% isotopic enrichment of A$\beta_{1\text{-}42}$, tryptic digestion was performed to further probe the protein sequence. However, the predominant species in the sample corresponds to 97-98% isotopically enriched E22G-A$\beta_{1\text{-}42}$.

Table S1: Monoisotopic masses for the tryptic digest fragment LVFFAGDVGSNK of E22G Aβ_{1-42}. In the B column, fragmentation is performed from N to C terminus while for fragmentation in the Y column fragmentation is performed from C to N.

Sequence	Sequence #	B	Y	Sequence #
L	1	120.825-121.088	1322.894-1325.609	12
V	2	225.664-226.154	1203.076-1205.528	11
F	3	382.355-383.219	1098.237-1100.463	10
F	4	539.046-540.284	941.546-943.397	9
A	5	613.929-615.318	784.855-786.332	8
G	6	673.834-675.336	709.972-711.298	7
D	7	793.662-795.361	650.067-651.280	6
V	8	898.502-900.425	530.239-531.256	5
G	9	958.407-960.444	425.400-426.191	4
S	10	1049.281-1051.473	365.494-366.172	3
N	11	1169.092-1171.510	274.621-275.143	2
K	12	1304.884-1307.598	154.809-155.106	1
Total Monoisotopic mass (M)		1321.887-1324.602	1322.662-1325.377	
$(M+2H)^{2+}$		661.951-663.308	662.338-663.696	

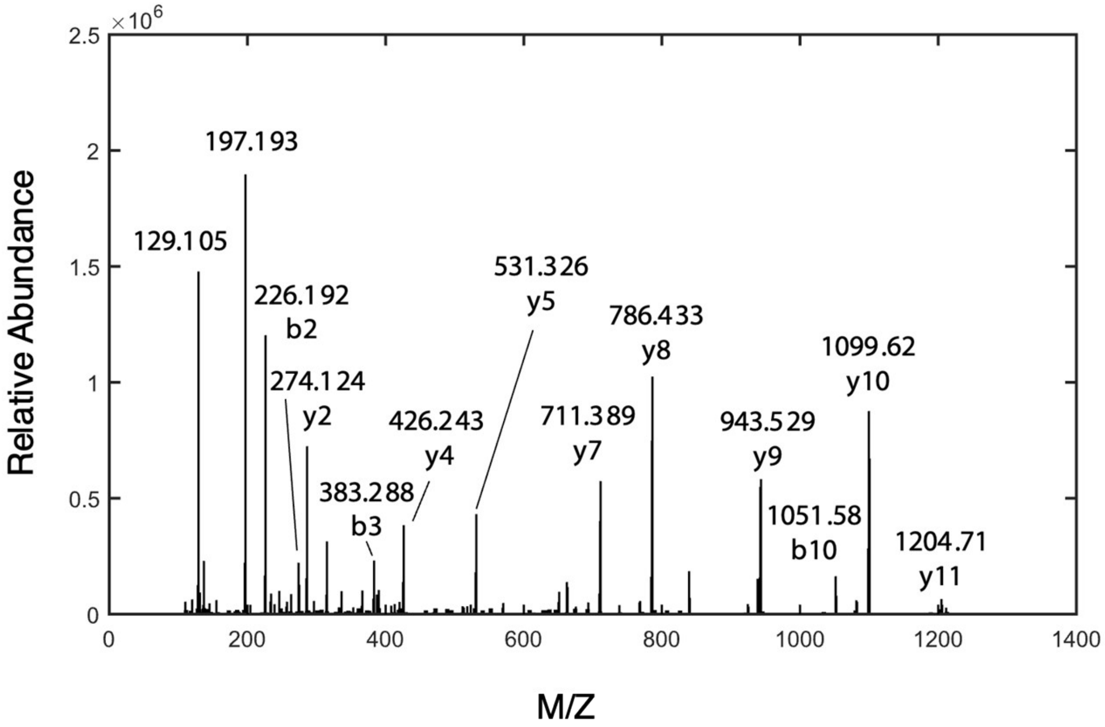

Figure S2: MS2 fragment spectrum of the 662.908 m/z peak. Overall, enough fragments are identified to confirm the E22G mutation was adopted and isotopically labeled at 97-100% enrichment. Tryptic digestion and subsequent fragmentation did not detect wild type A$\beta_{1\text{-}42}$.

Table S2: Acquisition parameters for MAS NMR experiments.

Spectrum	Max Evolution Time (ms)			Number of complex points			Spectral Width (Hz/ppm)			C-C Mixing Time (ms)	Scans per point	Interscan delay (s)	Experiment time
	ω_1	ω_2	ω_3	t_1	t_2	t_3	ω_1	ω_2	ω_3				
(H)NH GNNQQNY	19.7	15.9		160	1024		4050/50	32051/40		N/A	32	1.5	2 h 27 min
(H)CH GNNQQNY	16.3	12.56		460	2048		14069/70	81521/102		N/A	32	1.5	7 h 25 min
hCCH-RFDR GNNQQNY	5.3	5.3	10.2	128	128	2048	12059/60	12059/60	100,000/125	1.7	8	1.5	2d 14 h 57 min
hCC RFDR GNNQQNY	8.5	17.2		1024	2048		60000/298.5	59523/296.1		1.6	32	3	1d 3h 34 min
(H)NH E22G AB1-42	19.7	15.9		160	1024		4050/50	32051/40		N/A	32	1.5	2 h 27 min
(H)CH E22G $A\beta_{1\text{-}42}$	16.3	12.56		460	2048		14069/70	81521/102		N/A	32	1.5	7 h 25 min
hCCHRFDR E22G $A\beta_{1\text{-}42}$	5.3	5.3	10.2	128	128	2048	12059/60	12059/60	100,000/125	1.4	32	1.5	10d 11 h 47 min
hCCH-TOCSY E22G $A\beta_{1\text{-}42}$	5.3	5.3	10.2	128	128	2048	12059/60	12059/60	100,000/125	9	32	1.5	10d 12 h 53 min

Res	Cα	Cβ	Cγ1	Cδ1	Cδ2	Cε
L17		44.68	29.23		25.25	
F19	60.16					
D23		41.3				
S26	56.2	65.3				
N27	53.81	41.4				
K28	54.76	34.84		29.22		41.63
G29	48.21					
A30	49.13	21.03				
I31		44.65	27.14			
I32	59.98	41.29	27.05			
M35	54.75	36.25				
I41	60.137	39.76		13.73		
A42	52.42	19.62				

Table S3. Chemical Shifts assigned to E22G Aβ_{1-42} fibrils.

Res	Cα	Cβ	Cγ1	Cδ1	Cδ2	Cε
L17		44.62	28.6		25.24	
F19	59.86					
D23		40.88				
S26	54.96	65.97				
N27	52.76	42.57				
K28	54.69	34.85		29.62		41.8
G29	48.22					
A30	50.22	20.99				
I31		43.94		27.2		
I32	59.72	40.84	27.17			
M35	54.34	36.28				
I41	59.7	39.57		14.09		
A42	52.3	20.18				

Table S4. Chemical shifts of wild type Aβ_{1-42} fibrils. Published chemical shifts from Bahri, et al [1] and Colvin, et al [2-3] for wild type Aβ_{1-42} fibrils.

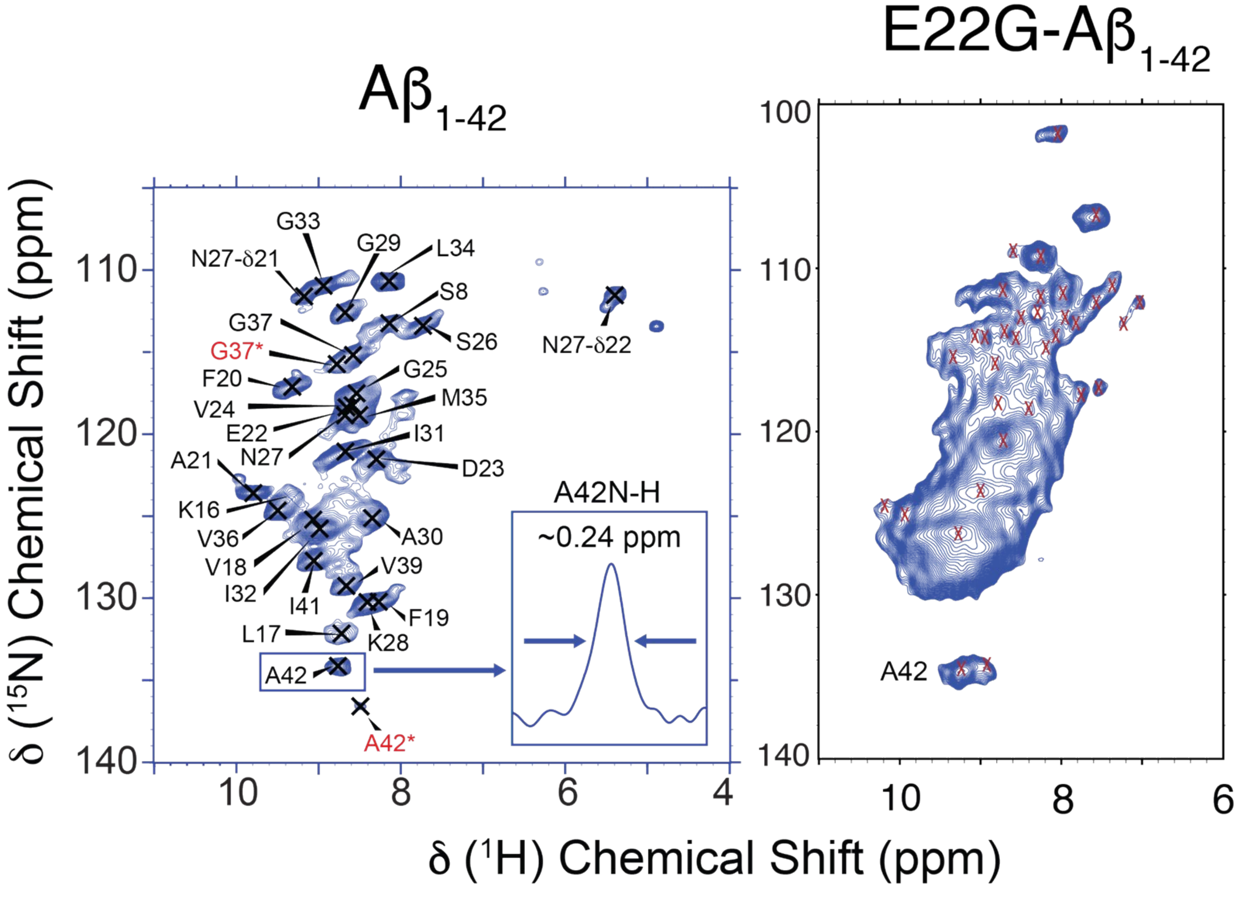

Figure S3: hNH correlation spectra of (left) $A\beta_{1-42}$ ($\omega_r/2\pi$=110 kHz, $\omega_{0H}/2\pi$ =1000 MHz) from Bahri,et al. (2022) [1] and (right) E22G-$A\beta_{1-42}$ ($\omega_r/2\pi$=90 kHz, $\omega_{0H}/2\pi$ =800 MHz). T=273K. Note the much improved resolution for the WT $A\beta_{1-42}$[1]. The “X’s” in the E22G-$A\beta_{1-42}$ spectrum denote possible cross peak positions.

(1) Bahri, S.; Silvers, R.; Michael, B.; Jaudzems, K.; Lalli, D.; Casano, G.; Ouari, O.; Lesage, A.; Pintacuda, G.; Linse, S.; Griffin, R. G., 1h Detection and Dynamic Nuclear Polarization–Enhanced Nmr of Aβ1-42 Fibrils, *Proc Natl Acad Sci USA* **2022**, *119*, e2114413119.

(2) Colvin, M. T.; Silvers, R.; Ni, Q. Z.; Can, T. V.; Sergeyev, I.; Rosay, M.; Donovan, K. J.; Michael, B.; Wall, J.; Linse, S.; Griffin, R. G., Atomic Resolution Structure of Monomorphic a Beta(42) Amyloid Fibrils, *J Am Chem Soc* **2016**, *138*, 9663-9674.

(3) Colvin, M. T.; Andreas, L. B.; Chou, J. J.; Griffin, R. G., Proton Association Constants of His 37 in the Influenza-a M218-60 Dimer-of-Dimers, *Biochemistry* **2014**, *53*, 5987-5994.
